# Supplementary material for: Design principles for NASICON super-ionic conductors
Source: Nat Commun. 2023 Aug 25;14:5210. doi: 10.1038/s41467-023-40669-0 (PMC10457403; doi:10.1038/s41467-023-40669-0)
Supplement: Supplementary file 1 — Supplementary Information [file 41467_2023_40669_MOESM1_ESM.pdf]

Design principles for NASICON super-ionic conductors

Supplementary information

Jingyang Wang[1,2,5], Tanjin He[1,2], Xiaochen Yang[1,2], Zijian Cai[1,2], Yan Wang[3], Valentina Lacivita[3], Haegyeom Kim[1], Bin Ouyang[4]* and Gerbrand Ceder[1,2]*

[1] Materials Sciences Division, Lawrence Berkeley National Laboratory, Berkeley, CA 94720, USA

[2] Department of Materials Science and Engineering, University of California, Berkeley, CA 94720, USA

[3] Advanced Material Lab, Samsung Advanced Institute of Technology and Samsung Semiconductor, Inc., Cambridge, MA 02138, USA

[4] Department of Chemistry and Biochemistry, Florida State University, Tallahassee, FL 32306, USA

[5] School of Sustainable Energy and Resources, School of Materials Science and Intelligent Engineering, Nanjing University, Suzhou, China

* Corresponding author:

Prof. Bin Ouyang (Email: bouyang@fsu.edu)

Prof. Gerbrand Ceder (Email: gceder@berkeley.edu)

Supplementary Table 1. Calculated energies and solid-state synthesis results of Na3-, sulfate-free NASICONs. *: electrochemically reported.

#	Composition	E_{hull}	1000K×S_{ideal}	Synthesized
1	*$Na_3Hf_2Si_2PO_{12}$	-5.23	18.23	
2	$Na_3HfTa(SiO_4)_3$	1.84	15.58	$NaTaO_3$ + HfO_2
3	$Na_3HfZrSi_2PO_{12}$	4.02	25.26	Y
4	$Na_3HfNb(SiO_4)_3$	6.79	15.58	$NaNbO_3$+$Na_2HfSi_2O_7$
5	*$Na_3MgZr(PO_4)_3$	7.27	15.58	
6	$Na_3HfMg(PO_4)_3$	7.54	15.58	Y
7	*$Na_3Al_2(PO_4)_3$	7.62	15.58	
8	$Na_3Hf_{1.5}Mg_{0.5}SiP_2O_{12}$	7.98	23.93	Y
9	$Na_3MgSn(PO_4)_3$	11.89	15.58	Precursors melt at ~600 °C
10	$Na_3TaSn(SiO_4)_3$	12.05	15.58	$NaTaO_3$+$Na_4Sn_2(SiO_4)_3$+SnO_2
11	$Na_3Mg_{1.33}Nb_{0.66}(PO_4)_3$	12.18	15.00	Precursors melt at ~600 °C
12	*$Na_3Zr_2Si_2PO_{12}$	12.20	18.23	
13	$Na_3Mg_{1.33}Ta_{0.66}(PO_4)_3$	12.99	15.00	Unindexed phases
14	$Na_3ScY(PO_4)_3$	14.56	15.58	Y
15	$Na_3HfScSiP_2O_{12}$	14.97	25.26	Y
16	*$Na_3ZrScSiP_2O_{12}$	15.12	25.26	
17	$Na_3HfSnSi_2PO_{12}$	16.06	25.26	NASICON phase+SnO_2
18	$Na_3Hf_{1.5}Ca_{0.5}SiP_2O_{12}$	17.09	23.93	Y
19	$Na_3ScIn(PO_4)_3$	17.11	15.58	Y
20	$Na_3Zr_{1.5}Mg_{0.5}SiP_2O_{12}$	17.43	23.93	Y
21	*$Na_3HfTiSi_2PO_{12}$	19.40	25.26	
22	$Na_3Zr_{1.5}Ca_{0.5}SiP_2O_{12}$	23.09	23.93	
23	$Na_3Hf_{1.5}Zn_{0.5}SiP_2O_{12}$	21.97	23.93	

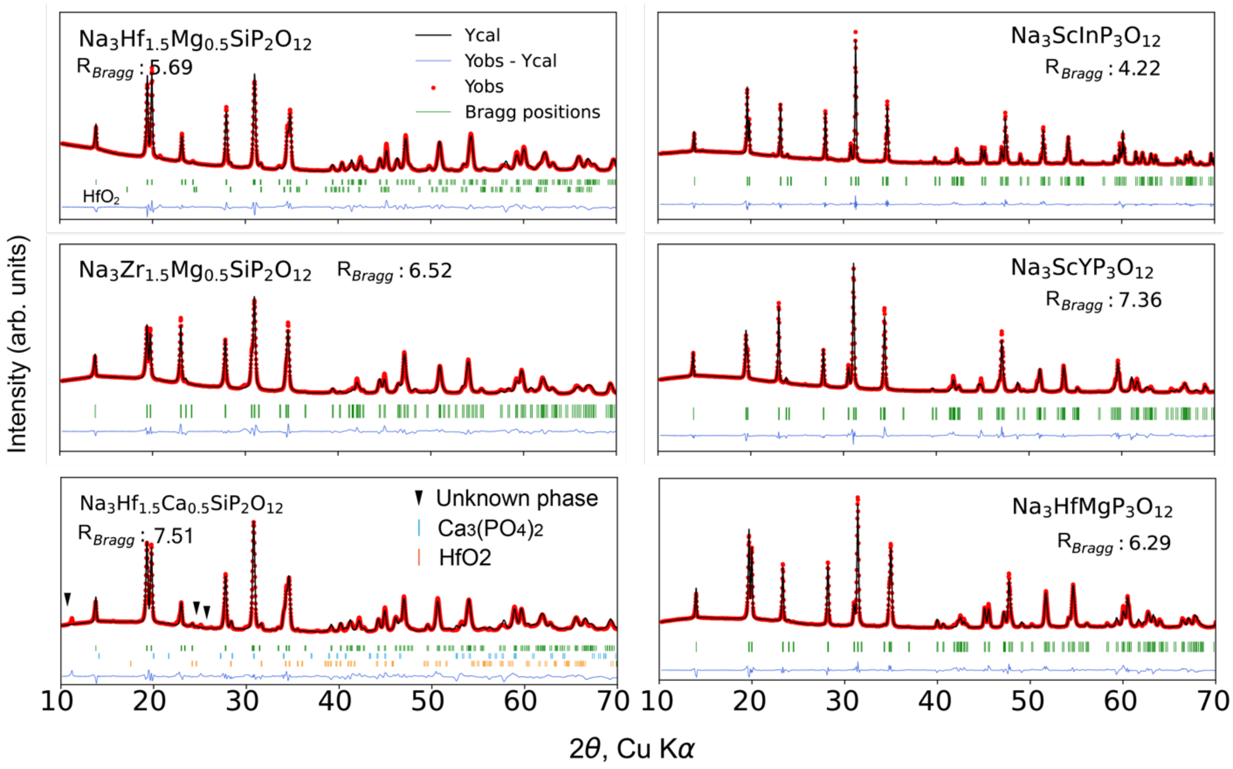

Supplementary Figure 1. Rietveld refinement results of 6 as-synthesized NASICONs. All refinements shown in this figure were performed with the rhombohedral *R-3c* space group. Note that $Na_3Hf_{1.5}Ca_{0.5}(SiO_4)(PO_4)_2$ contains 15.63 % of $Ca_3(PO_4)_2$ impurity, while all others are either phase pure or contain trace amount of HfO_2 impurity.

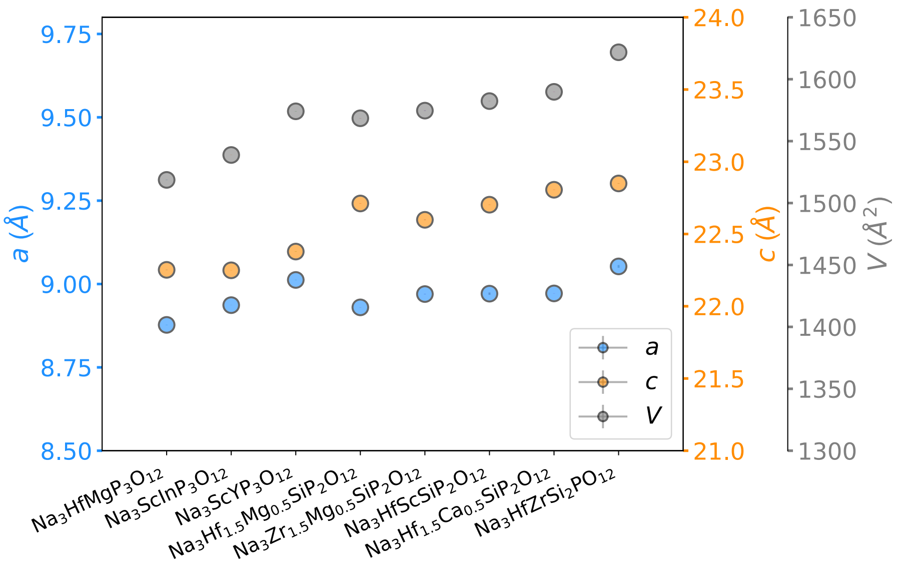

Supplementary Figure 2. Refined *a* (blue), *c* (orange) and volume (*V*, grey) lattice parameters of the as-synthesized NASICON in a rhombohedral cell.

Supplementary Table 2. Rietveld refinement structural parameters for $Na_3HfZrSi_2PO_{12}$.

Structural parameters for $Na_3HfZrSi_2PO_{12}$, wt (%):85.85 (3.92), R_{bragg} = 3.09 %, R_{wp} = 2.87 %, R_p = 2.14 %<br>Impurity: ZrO_2, wt % = 14.15 (0.78)					
S.G.	*a* (Å)	*b* (Å)	*c* (Å)	β (°)	*V* (Å^3)
C 2/c	15.67990 (416)	9.05675 (233)	9.23748 (171)	123.92139 (1270)	1088.541 (0.451)
Atom	site	x	y	z	Occupancy
O1	8f	0.15124	0.45308	0.26247	8.000
O2	8f	0.44510	0.45245	0.10529	8.000
O3	8f	0.25461	0.18107	0.21604	8.000
O4	8f	0.36899	0.12751	0.12364	8.000
O5	8f	0.43128	0.19546	0.41100	8.000
O6	8f	0.09044	0.14565	0.25827	8.000
P1/Si1	4e	0.00000	0.03353	0.25000	1.6/2.4
P2/Si2	8f	0.35209	0.10466	0.25313	3.2/4.8
Na1	4d	0.25000	0.25000	0.50000	2.774 (152)
Na2	4e	0.50000	0.87983	0.25000	4.000
Na3	8f	0.82210	0.09581	0.81885	4.602(406)
Hf	8f	0.09960 (84)	0.25185 (394)	0.05618 (54)	4.922 (209)
Zr	8f	0.09960 (84)	0.25185 (394)	0.05618 (54)	3.078 (209)

Refined composition: $Na_{2.845}Hf_{1.1231}Zr_{0.769}Si_{1.80}P_{1.20}O_{12}$

Supplementary Table 3. Rietveld refinement structural parameters for $Na_3HfScSiP_2O_{12}$.

Structural parameters for $Na_3HfScSiP_2O_{12}$, wt % = 98.65 (2.32), R_{bragg} = 2.94 %, R_{wp} = 4.85 %, R_p = 3.56 % Impurity: HfO_2, wt % = 1.35 (0.07)					
S.G.	*a* (Å)	*b* (Å)	*c* (Å)	*V* (Å^3)	
R-3c	8.97105 (35)	8.97105 (35)	22.70273 (116)	1582.323 (0.119)	
Atom	site	x	y	z	Occupancy
O1	36f	0.03515 (145)	0.21466 (115)	0.19699 (56)	6.000
O2	36f	0.19208 (116)	0.16613 (132)	0.08691 (63)	6.000
P	18e	0.29139 (89)	0.00000	0.25000	1.846
Si	18e	0. 29139 (89)	0.00000	0.25000	1.154
Na1	6b	0.00000	0.00000	0.00000	0.688 (24)
Na2	18e	0.62255 (196)	0.00000	0.25000	2.090 (38)
Hf	12c	0.00000	0.00000	0.14868 (10)	1.014 (16)
Sc	12c	0.00000	0.00000	0.14868 (10)	0.986 (16)

Refined composition: $Na_{2.778}Hf_{1.014}Sc_{0.986}Si_{1.154}P_{1.846}O_{12}$

Supplementary Table 4. Rietveld refinement structural parameters for $Na_3Hf_{1.5}Ca_{0.5}SiP_2O_{12}$.

Structural parameters for $Na_3Hf_{1.5}Ca_{0.5}SiP_2O_{12}$, wt (%): 83.10 (0.44), R_{bragg} = 7.25 %, R_{wp} = 5.09 %, R_p = 3.58 %

Impurities: $Ca_3(PO_4)_2$, wt % = 15.63 (0.35); HfO_2, wt % = 1.26 (0.07)

S.G.	*a* (Å)	*b* (Å)	*c* (Å)	*V* ($Å^3$)	
R-3c	8.97903 (18)	8.97903 (18)	22.82426 (67)	1593.625 (0.065)	
**Atom**	**site**	**x**	**y**	**z**	**Occupancy**
O1	36f	0.04418 (94)	0.22975 (87)	0.20216 (41)	6.000
O2	36f	0.19496 (89)	0.17859 (93)	0.08170 (38)	6.000
P	18e	0.30390 (82)	0.00000	0.25000	1.694
Si	18e	0.30390 (82)	0.00000	0.25000	1.306
Na1	6b	0.00000	0.00000	0.00000	0.632 (18)
Na2	18e	0.64515 (171)	0.00000	0.25000	2.194 (37)
Hf	12c	0.00000	0.00000	0.14779 (6)	1.728 (15)
Ca	12c	0.00000	0.00000	0.14779 (6)	0.272 (15)

Refined composition: $Na_{2.826}Hf_{1.728}Ca_{0.272}Si_{1.306}P_{1.694}O_{12}$

Supplementary Table 5. Rietveld refinement structural parameters for $Na_3Zr_{1.5}Mg_{0.5}SiP_2O_{12}$.

Structural parameters for $Na_3Zr_{1.5}Mg_{0.5}SiP_2O_{12}$, R_{bragg} = 6.52 %, R_{wp} = 4.70 %, R_p = 3.28 %					
S.G.	*a* (Å)	*b* (Å)	*c* (Å)	*V* (Å^3)	
R-3c	8.96578 (49)	8.96578 (49)	22.58554 (134)	1572.306 (0.099)	
Atom	site	x	y	z	Occupancy
O1	36f	0.02938 (89)	0.22068 (79)	0.19692 (30)	6.000
O2	36f	0.19283 (65)	0.16542 (71)	0.08423 (36)	6.000
P	18e	0.28935 (45)	0.00000	0.25000	2.156
Si	18e	0.28935 (45)	0.00000	0.25000	0.844
Na1	6b	0.00000	0.00000	0.00000	0.795 (10)
Na2	18e	0.63769 (107)	0.00000	0.25000	2.139 (17)
Zr	12c	0.00000	0.00000	0.14804 (8)	1.458 (8)
Mg	12c	0.00000	0.00000	0.14804 (8)	0.542 (8)

Refined composition: $Na_{2.934}Zr_{1.458}Mg_{0.542}Si_{0.844}P_{2.156}O_{12}$

Supplementary Table 6. Rietveld refinement structural parameters for $Na_3Hf_{1.5}Mg_{0.5}SiP_2O_{12}$.

Structural parameters for $Na_3Hf_{1.5}Mg_{0.5}SiP_2O_{12}$, wt % = 98.62 (1.40), R_{bragg} = 5.69 %, R_{wp} = 4.20 %, R_p = 2.82 %
Impurity: HfO_2, wt % = 1.08 (0.07)

S.G.	*a* (Å)	*b* (Å)	*c* (Å)	*V* (Å^3)	
R-3c	8.93224 (15)	8.93224 (15)	22.71948(51)	1569.818 (0.052)	
**Atom**	**site**	**x**	**y**	**z**	**Occupancy**
O1	36f	0.03185 (82)	0.22414 (77)	0.20098 (34)	6.000
O2	36f	0.19783 (75)	0.17173 (77)	0.08091 (32)	6.000
P	18e	0.29260 (56)	0.00000	0.25000	1.638
Si	18e	0.29260 (56)	0.00000	0.25000	1.362
Na1	6b	0.00000	0.00000	0.00000	0.767 (16)
Na2	18e	0.63595 (147)	0.00000	0.25000	2.154 (31)
Hf	12c	0.00000	0.00000	0.14753 (5)	1.717 (11)
Mg	12c	0.00000	0.00000	0.14753 (5)	0.283 (11)

Refined composition: $Na_{2.921}Hf_{1.717}Mg_{0.283}Si_{1.362}P_{1.638}O_{12}$

Supplementary Table 7. Rietveld refinement structural parameters for $Na_3HfMg(PO_4)_3$.

Structural parameters for $Na_3HfMg(PO_4)_3$, R_{bragg} = 5.11 %, R_{wp} = 3.19 %, R_p = 2.10 %					
S.G.	*a* (Å)	*b* (Å)	*c* (Å)	*V* (Å^3)	
R-3c	8.88030 (24)	8.88030 (24)	22.25834 (65)	1520.122 (0.074)	
Atom	site	x	y	z	Occupancy
O1	36f	0.02917 (62)	0.21711 (52)	0.19591 (19)	6.000
O2	36f	0.19090 (43)	0.17148 (48)	0.08416 (21)	6.000
P	18e	0.29374 (33)	0.00000	0.25000	3.000
Na1	6b	0.00000	0.00000	0.00000	0.847 (4)
Na2	18e	0.64113 (69)	0.00000	0.25000	2.216 (4)
Hf	12c	0.00000	0.00000	0.14756 (4)	0.969 (5)
Mg	12c	0.00000	0.00000	0.14756 (4)	1.031 (5)

Refined composition: $Na_{3.063}Hf_{0.969}Mg_{1.031}P_3O_{12}$

Supplementary Table 8. Rietveld refinement structural parameters for $Na_3ScIn(PO_4)_3$.

Structural parameters for $Na_3ScIn(PO_4)_3$, R_{bragg} = 4.22 %, R_{wp} = 4.02 %, R_p = 2.77 %					
S.G.	*a* (Å)	*b* (Å)	*c* (Å)	*V* (Å^3)	
R-3c	8.93881 (5)	8.93881 (5)	22.25549 (15)	1540.022 (0.016)	
Atom	site	x	y	z	Occupancy
O1	36f	0.03190 (84)	0.22552 (87)	0.19461 (29)	6.000
O2	36f	0.19278 (77)	0.17096 (75)	0.08090 (36)	6.000
P	18e	0.29686 (47)	0.00000	0.25000	3.000
Na1	6b	0.00000	0.00000	0.00000	0.798 (8)
Na2	18e	0.64091 (84)	0.00000	0.25000	2.237 (8)
Sc	12c	0.00000	0.00000	0.14971 (8)	1.024 (7)
In	12c	0.00000	0.00000	0.14971 (8)	0.976 (7)

Refined composition: $Na_{3.03}Sc_{1.024}In_{0.976}P_3O_{12}$

Supplementary Table 9. Rietveld refinement structural parameters for $Na_3ScY(PO_4)_3$.

Structural parameters for $Na_3ScY(PO_4)_3$, R_{bragg} = 7.36 %, R_{wp} = 4.71 %, R_p = 3.04 %					
S.G.	*a* (Å)	*b* (Å)	*c* (Å)	*V* ($Å^3$)	
R-3c	9.01517 (33)	9.01517 (33)	22.38286 (89)	1575.410 (0.103)	
Atom	site	x	y	z	Occupancy
O1	36f	0.17592 (67)	0.19173 (66)	0.41366 (32)	6.000
O2	36f	0.03571 (76)	0.23202 (77)	0.19621 (25)	6.000
P	18e	0.00000	0.29907 (43)	0.25000	3.000
Na1	6b	0.00000	0.00000	0.00000	0.849 (11)
Na2	18e	0.64502 (89)	0.00000	0.25000	2.151 (11)
Sc	12c	0.00000	0.00000	0.34689 (8)	1.038 (16)
Y	12c	0.00000	0.00000	0.34689 (8)	0.962 (16)

Refined composition: $Na_{3.0}Sc_{1.038}Y_{0.962}P_3O_{12}$

Supplementary Table 10. Synthesis and densification conditions. All pellets sintered for electrochemical tests are ~ 6 mm in diameter and ~ 1 mm in thickness.

Composition	Synthesis condition	Densification condition	Estimated relative density
$Na_3HfMgP_3O_{12}$	900 °C, 12 h, Air	700 °C, 16 h, Air	95.3%
$Na_3ScInP_3O_{12}$	1000 °C, 12 h, Air	850 °C, 16 h, Air	86.7%
$Na_3ScYP_3O_{12}$	1200 °C, 10 h, Air	750 °C, 48 h, Air	93.0%
$Na_3Hf_{1.5}Mg_{0.5}SiP_2O_{12}$	1000 °C, 12 h, Air	1000 °C, 16 h, Air	93.8%
$Na_3Zr_{1.5}Mg_{0.5}SiP_2O_{12}$	1000 °C, 12 h, Air	1000 °C, 16 h, Air	95.0%
$Na_3HfScSiP_2O_{12}$	1000 °C, 12 h, Ar	1000 °C, 16 h, Ar	92.5%
$Na_3Hf_{1.5}Ca_{0.5}SiP_2O_{12}$	1100 °C, 10 h, Air	1000 °C, 16 h, Air	87.0%
$Na_3HfZrSi_2PO_{12}$	1100 °C, 10 h, Ar	1100 °C, 16 h, Ar	94.9%
$Na_{3+x}Hf_{1-x}ZrSc_xSi_2PO_{12}$ (x = 0.2, 0.4)	1100 °C, 10 h, Ar	1200 °C, 10 h, Ar	95.0%

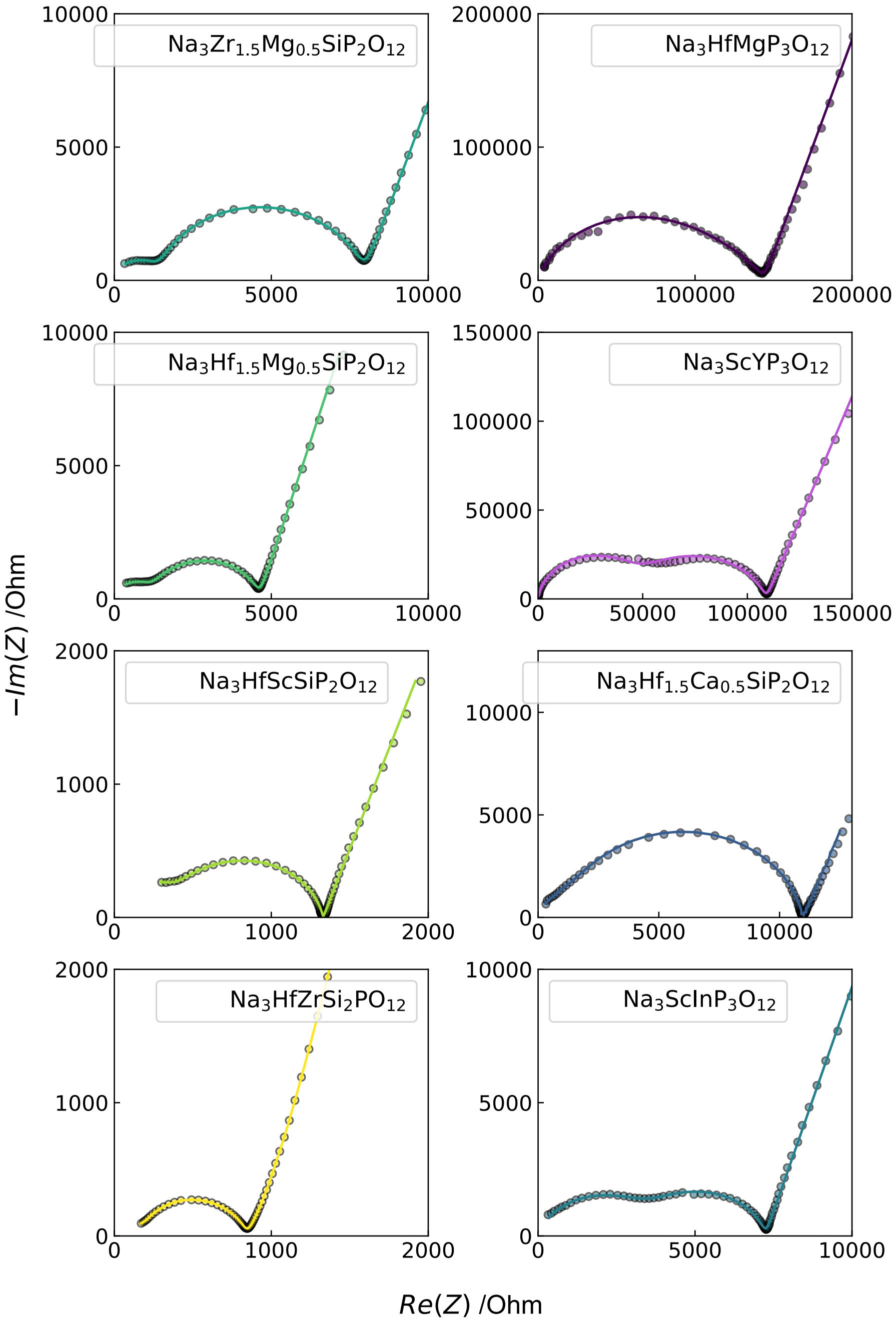

Supplementary Figure 3. Nyquist plots (scatter) taken at room temperature (~ 25 °C) and the corresponding fitting (line) of eight as-synthesized NASICONs. A theoretical circuit model with two R-C components (grain and grain boundary, respectively) were used for fitting except $Na_3HfZr(SiO_4)_2(PO_4)$ of which the bulk impedance was represented by a resistance, as shown in Supleementary Figure 4.

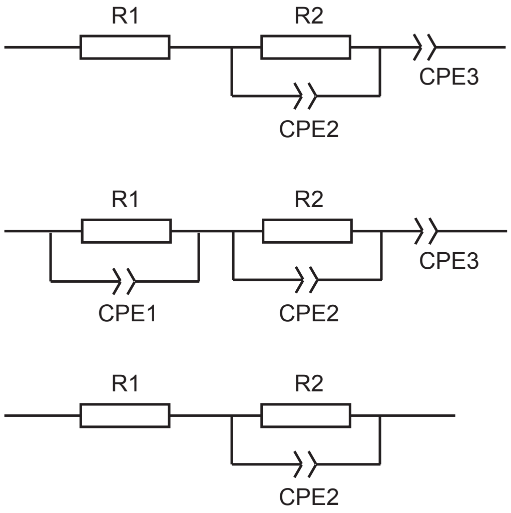

Supplementary Figure 4. Equivalent circuit models used for the fitings in Supplementary Figure 3 ($Na_3HfZr(SiO_4)_2(PO_4)$: top; others: middle), and Figure 5 (Sc00: top; others: bottom)

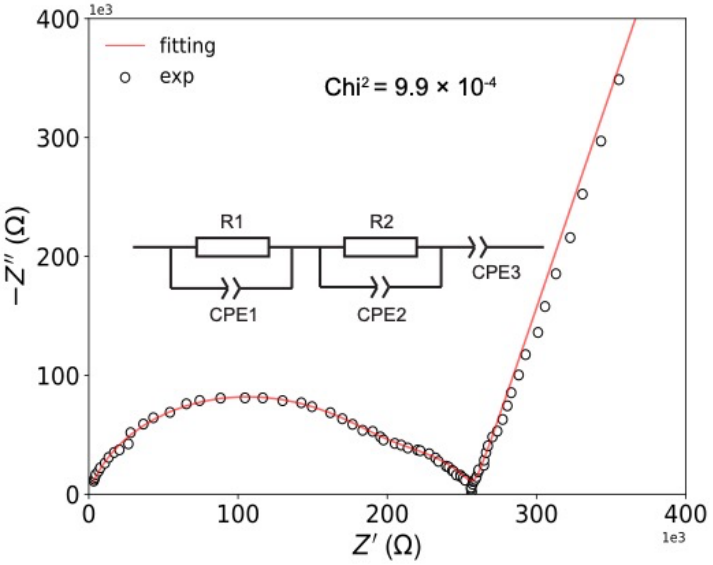

Supplementary Figure 5. Nyquist plots taken at 10 °C for $Na_3HfMg(PO_4)_3$. Inset shows the equivalent circuit model used for fitting with $Chi^2 = 4.76 \times 10^{-4}$

Supplementary Table 11. Fitting parameters for Nyquist plots shown in Supplementary Figure 3 and Figure 5.

		R1	CPE1-T	CPE1-P	Q1	R2	CPE2-T	CPE2-P	Q2	CPE3-T	CPE3-P
**HfZr**	Fit values	135.2	-	-	-	723	8.18×10^{-9}	0.8	4.06×10^{-10}	4.06×10^{-6}	0.82
$Chi^2 = 1.12 \times 10^{-3}$	error %	1.78	-	-	-	0.62	9.06	0.76		3.20	0.58
**HfSc**	Fit values	338.1	8.17×10^{-12}	1.09	4.09×10^{-11}	991.2	1.60×10^{-9}	0.88	2.79×10^{-10}	1.06×10^{-4}	0.79
$Chi^2 = 2.56 \times 10^{-4}$	error %	1.59	23.60	1.25		0.58	4.46	0.40		0.48	0.20
**HfMgSiP2**	Fit values	1121	8.53×10^{-11}	0.94	3.27×10^{-11}	3485	3.55×10^{-9}	0.86	5.26×10^{-10}	1.13×10^{-6}	0.83
$Chi^2 = 4.76 \times 10^{-4}$	error %	1.65	14.97	0.93		0.80	8.42	0.81		2.24	0.44
**HfMgP3**	Fit values	112430	1.17×10^{-10}	0.83	1.12×10^{-11}	30813	3.08×10^{-9}	0.82	4.03×10^{-10}	7.18×10^{-7}	0.82
$Chi^2 = 2.14 \times 10^{-3}$	error %	2.35	0.88	-		8.42	20.14	-		0.62	0.35
**ZrMgSiP2**	Fit values	1137	6.24×10^{-11}	0.96	3.07×10^{-11}	6580	2.70×10^{-9}	0.88	1.89×10^{-9}	1.33×10^{-6}	0.80
$Chi^2 = 2.88 \times 10^{-4}$	error %	0.88	9.48	0.58		0.37	4.56	0.42		0.70	0.19
**HfCaSiP2**	Fit values	1265	3.38×10^{-10}	0.88	4.68×10^{-11}	9700	5.12×10^{-10}	0.89	1.18×10^{-10}	3.99×10^{-5}	0.77
$Chi^2 = 9.4 \times 10^{-4}$	error %	4.98	19.93	1.37		0.72	6.20	0.59		0.62	0.41
**ScInP3**	Fit values	3419	6.20×10^{-10}	0.83	3.95×10^{-11}	3818	8.66×10^{-9}	0.84	1.14×10^{-9}	6.82×10^{-6}	0.81
$Chi^2 = 1.5 \times 10^{-3}$	error %	2.62	11.18	0.83		2.68	18.97	2.20		1.37	0.50
**ScYP3**	Fit values	45580	5.56×10^{-11}	0.95	2.82×10^{-11}	63069	5.67×10^{-9}	0.79	7.24×10^{-10}	8.29×10^{-6}	0.78
$Chi^2 = 4.26 \times 10^{-3}$	error %	3.34	0.25	0.74		2.92	19.18	2.96		1.46	1.24
**Sc00**	Fit values	169.5	-	-	-	683.2	4.37×10^{-9}	0.85	4.36×10^{-10}	1.61×10^{-6}	0.79
$Chi^2 = 7.1 \times 10^{-4}$	error %	1.42	-	-	-	0.79	10.72	0.84		4.68	0.73
**Sc02**	Fit values	93.16	-	-	-	517.6	2.28×10^{-9}	0.93	7.69×10^{-10}	-	-
$Chi^2 = 4.5 \times 10^{-3}$	error %	1.42	-	-	-	0.66	10.10	0.72		-	-
**Sc04**	Fit values	54.93	-	-	-	207.3	6.81×10^{-9}	0.90	1.61×10^{-9}	-	-
$Chi^2 = 4.5 \times 10^{-3}$	error %	1.95	-	-	-	1.08	2.22	0.13		-	-

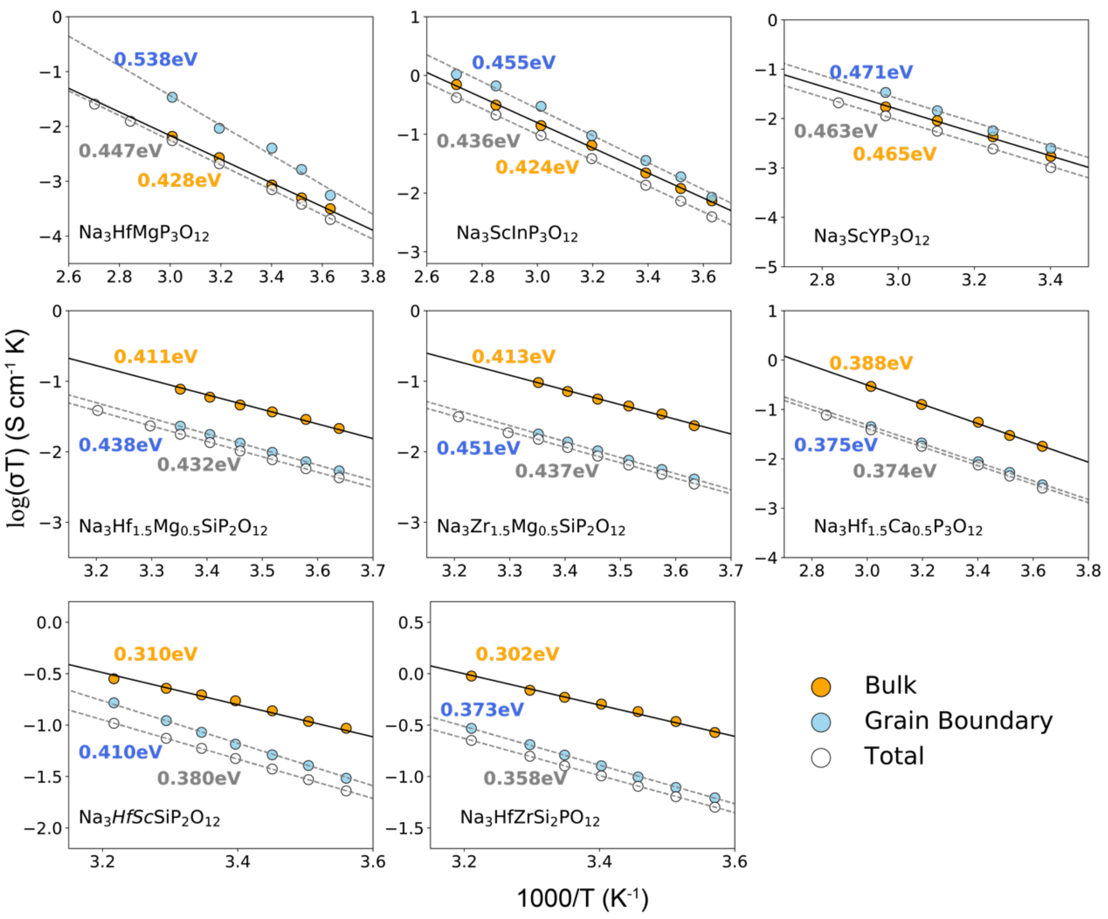

Supplementary Figure 6. Arrhenius plots and the corresponding linear fittings of the eight as-synthesized NASICONs. Bulk (orange) and grain boundary (light blue) contributions are fitted and plotted separately. For some high-temperature data of which the bulk and grain boundary contribution cannot be deconvoluted, only total conductivities were taken, as shown in the figure.

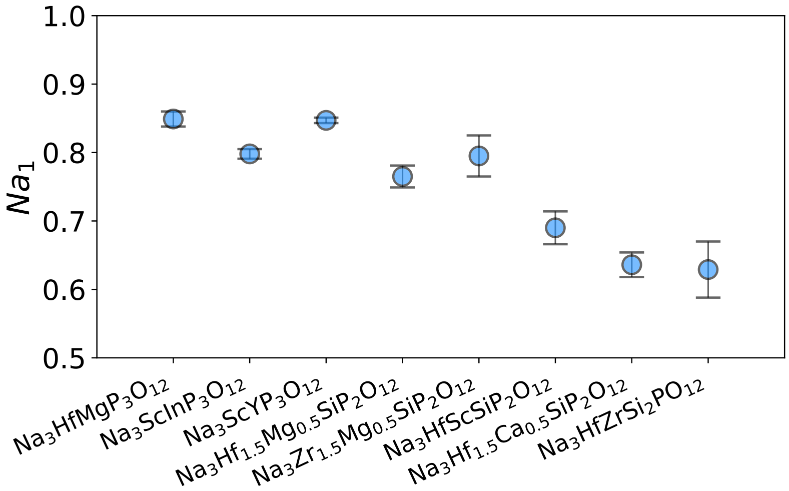

Supplementary Figure 7. Refined Na1 occupancy of the as-synthesized NASICONs.

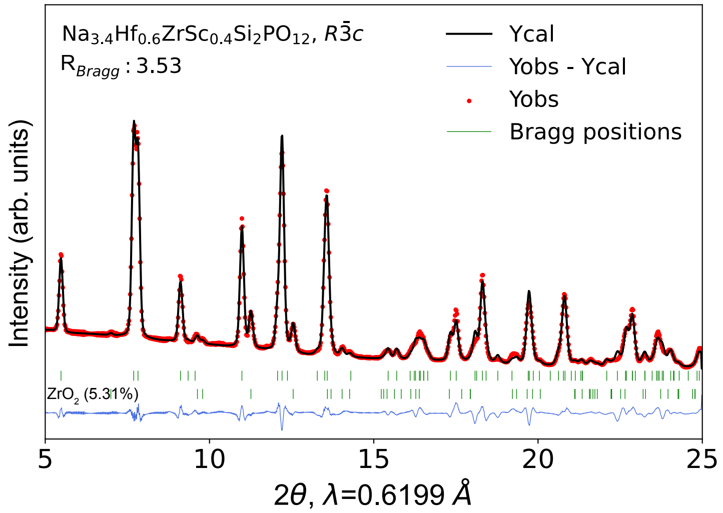

Supplementary Figure 8. Rietveld refinement results of the synchrotron XRD pattern of $Na_{3.4}Hf_{0.6}Sc_{0.4}ZrSi_2PO_{12}$. The structural parameters are summarized in Supplementary Table 12.

Supplementary Table 12. Rietveld refinement structural parameters for $Na_{3.4}Hf_{0.6}Sc_{0.4}ZrSi_2PO_{12}$.

Structural parameters for $Na_{3.4}Hf_{0.6}Sc_{0.4}ZrSi_2PO_{12}$, wt % = 94.69 (2.96), R_{bragg} = 3.53 %, R_{wp} = 3.10 %, R_p = 2.21%
Impurity: ZrO_2, wt % = 5.31 (0.19)

S.G.	*a* (Å)	*b* (Å)	*c* (Å)	*V* (Å^3)	
R-3c	9.07032 (49)	9.07032 (49)	22.78460 (152)	1623.370 (0.164)	
**Atom**	**site**	**x**	**y**	**z**	**Occupancy**
O1	36f	0.02744 (192)	0.21300 (163)	0.19714 (74)	6.000
O2	36f	0.18589 (137)	0.15674 (164)	0.08733 (62)	6.000
P	18e	0.28762 (112)	0.00000	0.25000	1.316
Si	18e	0.28762 (112)	0.00000	0.25000	1.684
Na1	6b	0.00000	0.00000	0.00000	0.711 (22)
Na2	18e	0.63554 (216)	0.00000	0.25000	2.429 (41)
Hf	12c	0.00000	0.00000	0.14815 (13)	0.700 (30)
Zr	12c	0.00000	0.00000	0.14815 (13)	0.845 (30)
Sc	12c	0.00000	0.00000	0.14815 (13)	0.455 (30)

Refined composition: $Na_{3.14}Hf_{0.700}Sc_{0.455}Zr_{0.845}Si_{1.684}P_{1.316}O_{12}$

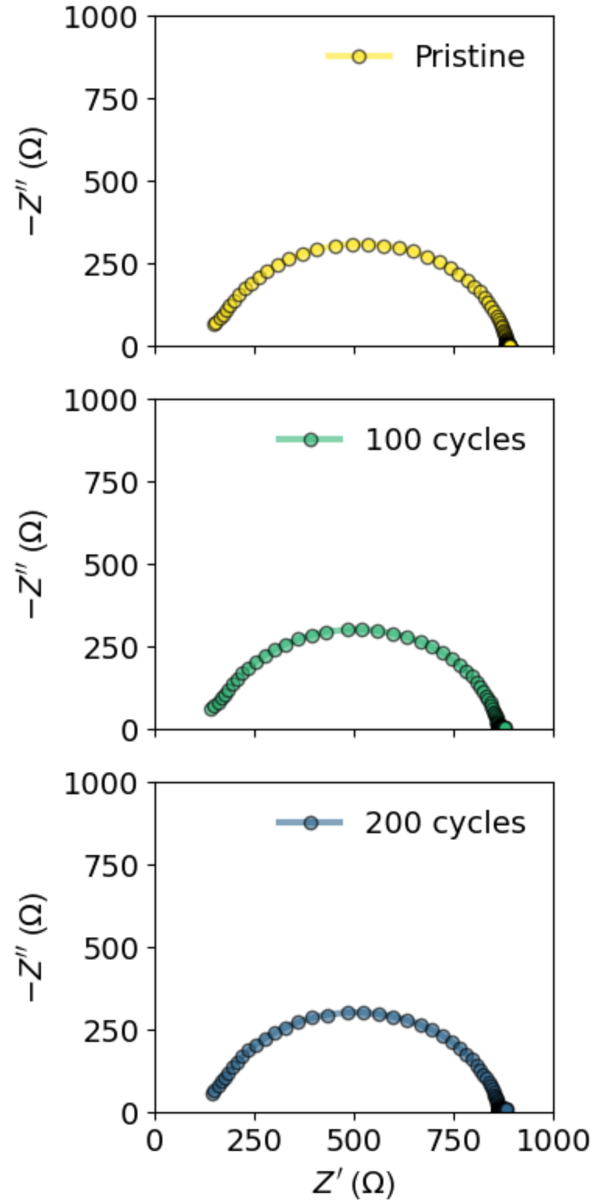

Supplementary Figure 9. Nyquist plots of a Na|$Na_{3.4}Hf_{0.6}Sc_{0.4}ZrSi_2PO_{12}$|Na symmetric cell at its pristine state (top), after 100 cycles at 0.1 mA cm^{-2} (middle) and after another 100 cycles at 0.2 mA cm^{-2} (bottom). All data are taken in a constant temperature chamber at 25 °C.

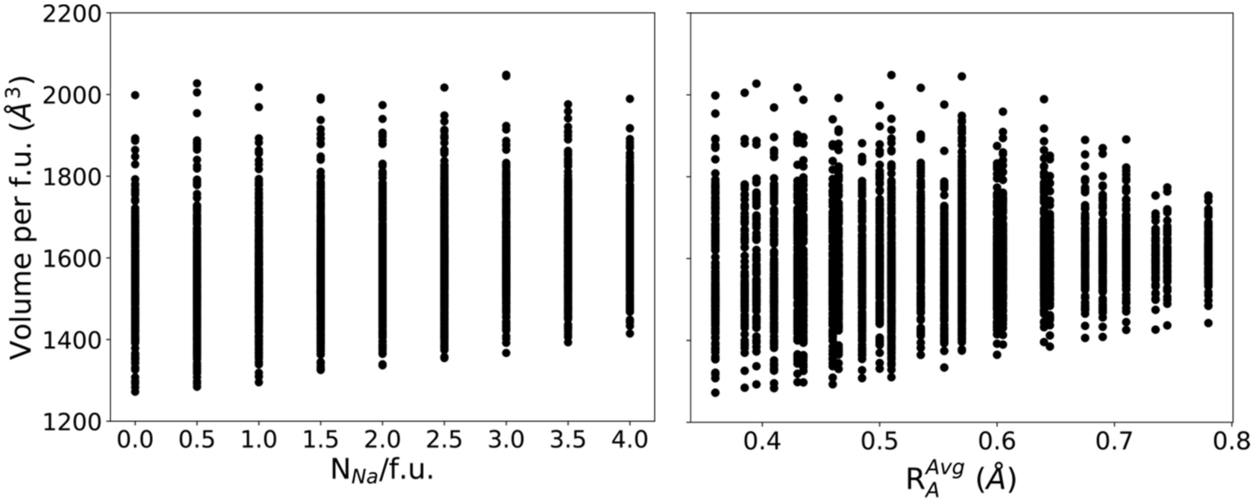

Supplementary Figure 10. Volume per formula unit as a function of Na content per formula unit (left), and average polyanion radius (right) for all calculated NASICONs. The volume per formula unit generally increases with larger Na content, but is disturbed by the variation of the size of *M*, *A* species.
